# Transparency of data on the value chain of medicines in Argentina, Brazil, and Colombia

**DOI:** 10.3389/fphar.2022.1063300

**Published:** 2023-01-05

**Authors:** Alane Andrelino Ribeiro, Angela Acosta, Marcela Amaral Pontes, Manuel Alejandro Machado Beltran, Rafaela Tavares Peixoto, Silvana Nair Leite

**Affiliations:** ^1^ Faculty of Health Sciences, University of Brasilia, Brasilia, Brazil; ^2^ Departamento de Ciencias Farmacéuticas, Facultad de Ciencias Naturales, Universidad ICESI, Cali, Colombia; ^3^ Faculty of Sciences, National University of Colombia, Bogotá, Colombia; ^4^ Department of Pharmaceutical Sciences, Federal University of Santa Catarina, Florianopolis, Brazil

**Keywords:** pharmaceutical policy, medicines, transparency, intellectual property, clinical trials, research and development, pricing

## Abstract

**Introduction:** The transparency of data on the value chain of medicines is crucial for the study and monitoring of the pharmaceutical system. These data may impact medicine pricing negotiations, contribute to patient access to pharmaceutical products, and strengthen health systems.

**Objective:** This study analyzed the national strategies to ensure the transparency of data from medicine cost development to marketing in Argentina, Brazil, and Colombia.

**Method:** A descriptive study was carried out by searching databases, reports, documents, and scientific articles published between January and August 2022 related to rules on transparency and databases, including 1) marketing authorization; 2) pricing; 3) intellectual property; 4) clinical trials; 5) research and development (R&D); and 6) health technology assessment (HTA) of selected biopharmaceuticals.

**Results:** Transparency data, rules, and information are not uniform. The infostructures (organizational capacity for collecting and distributing information) regarding the pharmaceutical value chain in these three countries face limitations in appropriate measures to publicly share data and evidence, including pre-clinical data, clinical data, and costs. None of the countries require transparency about data on research and development costs. All three countries present similar publicization of data on marketing authorization and intellectual property, with some differences. The significant limitations in Argentina include the absence of formal price regulation and data on the volume of medicine purchased and respective amounts paid. Among the three countries, Brazil showed a higher degree of information transparency, perhaps due to the legal regulation that guarantees citizens access to information of public interest. Brazil also stands out in terms of the public availability of HTA reports and pricing, in addition to the highest volume of information. In contrast, Colombia has in place a decree that allows 5 years of trial data exclusivity for new medicines, an act contrary to data transparency. Despite the different stages of transparency, no country has evidenced a robust use of these data in public policy decision-making.

**Conclusion:** The results reinforce the presence of information asymmetry between stakeholders, data fragmentation, data gaps and overlap, and difficulty in comparing available data across the three countries and the use of these data nationally to produce evidence.

## Introduction

The pharmaceutical system^1^ comprises research and development and innovation, manufacturing, marketing authorization, health technology assessment, pricing and reimbursement, procurement and supply, prescribing, dispensing, use, and pharmacovigilance. This value chain crosses the entire financial medical industrial complex (Basile and Feo, 2021). There is growing recognition of the important role of data transparency to better align this value chain to the access and rational use of medicines and in power relationships between stakeholders (Årdal et al., 2021; Vargas et al., 2022). A major challenge of such transparency is the availability of detailed instruments to support the disclosure of data and information on the value chain to analyze whether transparency aids these goals in an accessible and structured way throughout the entire chain. The OECD report on the role of transparency in avoiding a COVID-19-induced food crisis underscored that “transparency is not automatic: it requires investments in gathering comparable information, monitoring market and policy developments, and communicating clearly about the findings” (OECD, 2020).

The WHO’s Roadmap for Access to Medicines, Vaccines and Other Technologies noted the pressing need for unbiased information on health products, including prices and its status as an essential component of evidence-based decision-making to support expansion or changes in national lists of essential medicines (WHO, 2019). Concerns about the need to ensure universal access to COVID-19 health technologies, particularly those under development, have prompted additional calls for cost and price transparency (Transparency International, 2021).

In addition to what is stated in World Health Assembly (WHA) Resolution 72.8, transparency along the value chain must include transparency on net/real prices to increase buyers’ negotiating leverage by reducing information asymmetry, reducing prices, assessing pricing fairness, providing public accountability and trust, and understanding the market. Moreover, transparency on net/real R&D and manufacturing costs is needed to assess the fairness of prices, understand innovation systems, calibrate R&D incentives, understand manufacturing challenges and possibilities, provide transparency on revenues and units sold, demonstrate how marketing costs can be used to assess the fairness of prices, and understand the market. Moreover, transparency on clinical trial protocols and outcomes can promote patient safety, health and ethical treatments, assessment of the fairness of prices, calibration of prices for value for patients and health systems, and build public trust in products. In addition, transparency should be considered for patent licensing and other market exclusivities to understand where, for how long, and how strong a monopoly is in effect; the innovation system and information on licensing and various market exclusivities; and transparency on marketing authorization to identify the challenges and possibilities for availability; varying national assessment of safety and efficacy; and market links to the regulatory system (data exclusivity) (WHO, 2021a).

The Fair Pricing Forum 2021 report identified concrete measures to improve national transparency initiatives, including improving data collection and analysis in the public domain; persuasion, voluntary initiatives, and political pressure; ad hoc dissemination; access to information laws; and economic, regulatory, legislative, or judicial adjustments (WHO, 2021a; WHO, 2021b). To this end, understanding the influence of all stakeholders (such as States and other financial actors, industry, scientific and academic sectors, civil society organizations, and the international community) is needed to understand transparency relationships in the context of the pharmaceutical market. Global, regional, and national platforms for harmonized/standardized information sharing and the standardization of data reporting and sharing must be combined with accountability to citizens to explain how a certain price was arrived at and the obstacles faced by peripheral countries. These issues are intertwined with important challenges including information and data gaps; confidential negotiations and agreements that prevent price disclosure (Vogler and Paterson, 2017); conflict of interests; lack of guidance on reporting and comparing prices and related information; diversity of country contexts; unequal innovation systems; uncertainties; complexity of technology transfer, especially of biopharmaceuticals; barriers in innovation; and production capacity in peripheral countries (Vargas et al., 2022).

These issues are intensified in the context of Argentina, Brazil, and Colombia, three populous countries in South America, where there are deep endemic inequalities, technological and import dependence on biopharmaceutical products and active pharmaceutical ingredients, and other obstacles such as pricing and government budgets (Vargas et al., 2022). To date, little quantitative evidence has been reported related to the transparency of the pharmaceutical market and policies in South America (Lopes et al., 2022). Moreover, transparency levels have not been determined. Argentina has a Bismarckian healthcare model composed of three sectors: social insurance (*Obras Sociales*), private health insurance, and public health insurance. Healthcare in Argentina is mostly publicly financed, with some private insurance and out-of-pocket payments. Brazil has its Universal Health System (*Sistema Único de Saúde*—SUS), with complementary private participation. The Colombian healthcare system is mixed (*Sistema General de Seguridad Social en Salud*) and is funded primarily *via* public taxation with a notable role for private health insurance and out-of-pocket spending. Healthcare funding in the Colombia system is based on a contributive principle in which citizens with higher incomes and their employers subsidize the poorer strata of the population. The Colombian Ministry of Health publishes open data and information systems catalogs related to the national health system (Colombia productiva, 2022).

Brazil has the most predominant pharmaceutical supply chain among the three countries. In 2021, the pharmaceutical market in Argentina exported USD 884 million and reported USD 10,472 million in sales to the domestic market (CILFA, 2022). In contrast, the Brazilian pharmaceutical market is valued at 85.9 billion reals, with 7.9% growth from 2018 to 2019 (Brazil, 2021). The Colombian pharmaceutical market is valued at 14.5 billion pesos in 2018, with 7.6% growth from 2014 to 2018 (Colombia Productiva, 2022).

Latin American countries experience shortages and failures, including a “lack of linked data and cross-validated secondary data” (Leal et al., 2022) and “transparent, feasible, affordable, and protocol-driven data” (Lopes et al., 2022) on data sources related to quality, safety, purchases, medication use, and treatment adherence (Acosta et al., 2018; Lopes et al., 2022; Leal et al., 2022). This context may worsen due to the imminent recession and drastic increases in unemployment, poverty, and despondency.

To address these questions, we performed a comparative analysis of the transparency of data and information on the pharmaceutical systems in Argentina, Brazil, and Colombia to broaden the view and determine a set of hypotheses regarding barriers, feasibility, and options for the implementation of transparency policies for the pharmaceutical market in the regional context. These findings are also intended to strengthen the exchange of information between different initiatives and experiences and influence the development of policies both in the area of public health and in the field of economic regulation of the pharmaceutical industry.

## Methods

This descriptive study searched databases, reports, documents, and scientific articles between January and August 2022. For the selection and description of the results, we focused on six topics related to transparency elements in the pharmaceutical market.

The topics selected in this study were based on the “National transparency checklist on medicines and health products” published in August 2019 by the *Observatoire de la transparence dans les politiques du medicaments* (Mediapart, 2020). The checklist questions were adapted for six topics: 1) marketing authorization; 2) pricing; 3) intellectual property; 4) clinical trials; 5) research and development (R&D); and 6) health technology assessment (HTA). Each topic is divided into items that each represents one data type.

The data sources were padded using a multiphase approach including searches of institutional government websites, traditional bibliographic databases, and experts’ input. The reviewers independently searched, screened, and selected the data sources. Disagreements were resolved by consensus.

First, through a scoping review and documental recollection, the rules on transparency and databases were identified in the selected countries. Rules of transparency were defined as every rule expressed in acts, guidelines, bylaws, or any other enacted norm which made mandatory for companies, clinical trial organizations, universities, and or funders to disclose any of the data types in the registry, post-market approval, patent grant, incorporation on healthcare lists, or procurement. Database was defined as data types accessible by the public or regulators. Each database was investigated through form mapping and data dictionaries or manuals, if available, to identify all information available to the public or public officers.

We then compared transparency aspects between the medicines infliximab, trastuzumab, rituximab, tocilizumab, aflibercept, and etanercept in Argentina, Brazil, and Colombia (Tables 1, 2). These medicines include biological and biosimilar compounds and are marketed in all three countries. According to the CMED annual statistical report, trastuzumab and infliximab are among the top 10 revenue-producing medicines in Brazil. Due to their high prices, data and information transparency is an impact factor in public spending on health, market growth, complexity of R&D, and production. Furthermore, the high prices of these technologies have limited access by the population, especially considering the barriers to the entry of biosimilars into the market. Only robust, standardized, comparable information or databases were considered transparent and accessible in the present analysis. The promotion of sources of public information can allow the identification of trends, detection of differences, and comparisons and associations that can inform medicines policies in this region.

**TABLE 1:** Marketing authorization for infliximab in Argentina, Brazil and Colombia.

Country	Argentina	Brazil	Colombia
Marketing authorization	Four active records	Nine active records, tree of them canceled/expired	Two records, one in the process of renewal
Clinical trials information	No data on clinical trials are available in the technical advice	No data on clinical trials are available in the opinion	No data on clinical trials are available in the opinion
Research & development costs	No data on R&D costs are available in the technical advice	No data on R&D costs are available in the opinion	No data on R&D costs are available in the opinion
Complementary information	Includes registration number, laboratory, trade name, generic name, pharmaceutical form, presentation, retail price, API, GTIN, package insert and patient information. No technical advice on the granting of registration is available	All technical advice was granted. Includes a summary of clinical studies with their identification codes and conclusions on benefits and risks. It includes data from API, medicine, packaging and import manufacturers. The health record is also published in the official federal gazette by means of a Resolution	The marketing authorization does not include a summary of clinical studies, the conclusions on benefits and risks can be consulted with difficulty in the public minutes of the Evaluation Committee. It includes data from API, medicine, packaging and import manufacturers. On the website it is possible to consult the approved indications, pharmacological standard, contraindications, indications, interactions, pharmaceutical form, route of administration, storage conditions, and ATC

**TABLE 2:** Publicly accessible online registries of price and cost information in Argentina, Brazil, and Colombia.

Country	Attribute	Institution	Web portal	Taxonomy	Type of data/information
Argentina	Marketing authorization	*Administración Nacional de Medicamentos, Alimentos y Tecnología Medica* (ANMAT)	*Vademecum Nacional de Medicamentos* (VNM) http://anmatvademecum.servicios.pami.org.ar/index.html Warmings and recalls http://www.anmat.gov.ar/Medicamentos/Autorizaciones_VNM.asp	Open data/Open access/Restricted access/	Number of registration, name of the medicine, name of manufacturers. IFA, GTIN, leaflet and patient information. No technical advice on the approval of marketing authorization is available
					List of medicines unavailable in the country and their suppliers
	Pricing	Pharmaceutical sector platform	*Kairos web* https://ar.kairosweb.com/principio-activo/	Open data, volunteer initiative/private initiative	Private initiative global pricing, global price, name or the medicine, number of contracts. Pharmacy Retail Price (PRP)
		ANMAT	*Vademecum Nacional de Medicamentos* (VNM) http://anmatvademecum.servicios.pami.org.ar/index.html	Open government data, volunteer initiative	E-mail ANMAT License holders report the last Pharmacy Retail Price (PRP)
		Ministry of Health	List of reference prices 2022 (Resolution 27 de 2022) https://www.argentina.gob.ar/salud/seguimiento-precios/2022	Open government data	The Health Minister public monthly pricing list for chronic diseases Reference Price System (RPS)
		Ministry of Health	Analysis of the variation in medicine prices https://www.argentina.gob.ar/salud/seguimiento-precios/2021	Open government data	Available monthly analysis for two recently years (2020, 2021)
	Procurement prices	Government Public Procurement Portal	https://comprar.gob.ar/	Restricted access	Not available
	Reimbursement policies	Ministry of Health	List of reference prices 2022 (Resolución 27 de 2022) https://www.argentina.gob.ar/salud/seguimiento-precios/2022	Open government data	The Health Minister public monthly pricing list for chronic diseases Reference Price System (RPS)
	Patent information	*Instituto Nacional de la Propiedad Industrial* - INPI	https://portaltramites.inpi.gob.ar/PatenteConsultas/BusquedaParametros	Open access	IP database status of ongoing requests forwarded by the INPI for analysis of prior consent; status of each patent
	Clinical trial information	ANMAT	*Investigaciones clínicas farmacologicas* https://www.argentina.gob.ar/anmat/regulados/investigaciones-clinicas-farmacologicas and http://www.anmat.gov.ar/aplicaciones_net/applications/consultas/ensayos_clinicos/Consulta_EC.asp	Open access	Clinical trials database consultation of clinical trials authorized and not authorized by ANMAT; Its possible to download each ANMAT decisión which describe data and other issues, but doesn´t link clinical trials Id or protocol ID
			*Registro Nacional de Investigaciones en Salud* (*ReNIS*)*:* National Registry of Health Research https://www.argentina.gob.ar/salud/registroinvestigaciones and https://sisa.msal.gov.ar/sisa/#sisa		
					Study design, sponsor, registration number, funding source, population, contact information, status
		*Instituto de Efectividad Clínca y Sanitaria—IECS*	https:// www.iecs.org.ar/	Restricted access and data	Non-profit organization that creates HTA reports as needed in Argentina. Creates HTA reports as needed in Argentina
	Information on R&D expenditures	Not available	Not available	Restricted access and data	Not available
	HTA	*Comisión Nacional de Evaluación de Tecnologías de Salud* (CONETEC)	https://www.argentina.gob.ar/salud/conetec#:∼:text=La%20Comisi%C3%B3n%20Nacional%20de%20Evaluaci%C3%B3n,cobertura%20de%20las%20tecnolog%C3%ADas%20sanitarias	Under implementation	List of technologies prioritized for evaluation; analyses clinical and cost effectiveness; procures clinical guidelines and recommendations
		*Instituto de Efectividad Clínica y Sanitaria - IECS*	https:// www.iecs.org.ar/	Open access	Non-profit organization that creates HTA reports as needed in Argentina. Creates HTA reports as needed in Argentina
		*Unidad Coordinadora de Evaluación y Ejecución de Tecnologías en Salud (UCEETS)*	https://www.argentina.gob.ar/salud/instituto-nacional-del-cancer/coordinacion-investigacion/ets	Open access	Coordinates HTA activities across various agencies and act as a catalyst for the use of HTA in decision-making at the local and regional levels. Resolution 458/2009
Brazil	Marketing authorization	*Agência Nacional de Vigilância Sanitária* (Anvisa)	https://consultas.anvisa.gov.br/#/medicamentos/	Open access	Technical public advice on granting and refusing the registration (does not report cost data). Includes summary of clinical studies with respective identification codes and conclusions about benefits and risks. Includes data from IFA, drug, packaging and import manufacturers. medicine package insert
			Warnings and recalls https://sad.anvisa.gov.br/MicroStrategy/servlet/mstrWeb		
					Data on definitive or temporary discontinuation of medicines data on reactivation of manufacture and import of medicines
	Pricing	*Câmara de Regulação do Mercado de Medicamentos* (CMED)	https:// www.gov.br/anvisa/pt-br/assuntos/medicamentos/cmed	Restricted access/Restricted Data access/Open data	List of products with maximum selling price, list of medicines, list of medicines financed by the public system (Rename) list of oncological medicines. Public Maximum selling price; maximum wholesale prices; purchase prices of medical supply; the amounts. Details the price per product and per tax bracket. Identifies whether or not the product was marketed in the last year in the country
	Procurement prices	Official Gazette, Transparency Portal; Health Price Database Panel of prices of the Ministry of Economy	Official Gazette (*Diário Oficial da União*) https:// www.gov.br/imprensanacional/pt-br	Open access	Resolution inclusion in the CAP list, incorporation ordinance, extract of amendment, extract of contract. In which only the global value of the installment is available, the individual value or quantity is not available
			*Portal da Transparência* https://www.portaltransparencia.gov.br/ and https://www.portaltransparencia.gov.br/pagina-interna/603603-notas-fiscais	Open access	Contract details, value and quantity are provided
			*Banco de preços em Saúde* http://bps.saude.gov.br/login.jsf	Open access	The entire contract value, The quantity bought; public purchase data, contract data, Timetables for delivery; manufacturer/supplier data, institution data and values; Mean, median, maximum price, minimum price
			*Painel de preços Ministério da Economia* https://paineldeprecos.planejamento.gov.br/	Open access	The entire contract value, The quantity bought; public purchase data, contract data, Timetables for delivery; manufacturer/supplier data, institution data and values; Mean, median, maximum price, minimum price
	2.3 Reimbursement policies	Ministry of Health	Database TABNET^a^ http://tabnet.datasus.gov.br/cgi/tabcgi.exe?sia/cnv/qauf.def ^a^The total amount transferred to each territory is published as an ordinance in the official federal gazette	Open data	Approved amount, approved quantity, quarterly payment of state/municipal financing drugs with federal reimbursement
		Ministry of Health	Database SIGTAP http://sigtap.datasus.gov.br/tabela-unificada/app/sec/inicio.jsp	Open data	Value of the procedure (isolated drug, or the procedure linked to other services)
		Official Gazette	https://www.in.gov.br/servicos/diario-oficial-da-uniao	Open data	Value of the contract between government and pharmaceutical company
	3–Patent information	*Instituto Nacional de Propriedade Industrial* (INPI)	htps:// www.gov.br/inpi/pt-br/servicos/patentes t	Open access/legislation	IP database status of ongoing requests forwarded by the INPI for analysis of prior consent; status of each patent
		*Agência Nacional de Vigilância Sanitária* (Anvisa)	https://www.gov.br/anvisa/pt-br/setorregulado/regularizacao/medicamentos/propriedade-intelectual	Open access	End of prior consent by Anvisa
	Clinical trial information	*Agência Nacional de Vigilância Sanitária* (Anvisa)	https:// www.gov.br/anvisa/pt-br/assuntos/medicamentos/pesquisaclinica/ensaios-autorizados	Open data	Clinical trials database consultation of clinical trials authorized, canceled and not authorized by Anvisa; It’s possible to consult by disease, drug, ID protocol, period and status. It’s not possible to download each ANVISA decisión
		*Comissão Nacional de Ética em Pesquisa* (Conep)	https://portal.plataformamaisbrasil.gov.br/maisbrasil-portal-frontend/	Open data	Principal researcher, institution, research title and keywords. ^a^Approved clinical trials for Covid-19 Protocol, Company, Drug, Phase, Study Title, Database registration, expected completion, number of participants
		4.3 Registry of Clinical Trials (ReBEC)	4.3 https://ensaiosclinicos.gov.br/	4.3 - Open data	Research identification data, interventions, outcomes, study type, recruitment and contacts
	Information on R&D expenditures	Transparency´s portal	Portal da Transparência https://www.portaltransparencia.gov.br/	Open data	Only aggregated information about all public funding for R&D in Brazil
	HTA	Ministry of Health	The decisions are available on: http://conitec.gov.br/decisoes-sobre-incorporacao-ordem-alfabetica#T	Open access	Technical documents with the recommendation, decision of the committee, the committee meetings are recorded and made available online. List of proposed demands including requesting institution/company, flow of incorporation of technologies, public consultations and surveys. Status of development of clinical protocols and guidelines
		*Agência Nacional de Saúde Suplementar* - Cosaude	6.2–https://www.ans.gov.br/index.php/planos-de-saude-e-operadoras/espaco-do-consumidor/737-rol-de-procedimentos and http://www.ans.gov.br/participacao-da-sociedade/comites-e-comissoes/comite-permanente-de-regulacao-da-atencao-a-saude-cosaude	Open access	Terminologies, flows, list of technologies, technical opinions, clinical guidelines, committee members, meeting minutes and documents
		Brazilian Health Technology Assessment Network - REBRATS	https://rebrats.saude.gov.br/quem-somos	Open access	Integrates Health Technology Assessment Centers - NATS (public or private non-profit institutions, resources and professionals with technical competence to develop, promote and execute HTA)
Colombia	1–Marketing authorization	*Instituto Nacional de Vigilância de Medicamentos y Alimentos* (Invima)	https:// www.invima.gov.co/	Open data	Number of registration, name of the medicine, name of manufacturers. IFA, GTIN, leaflet and patient information. There is no technical document on the granting of registration
	2.1–Pricing	*Ministerio de Salud y Protección Social*	https://www.sispro.gov.co/Pages/Home.aspx	Restricted data	Pricing database
			https://www.minsalud.gov.co/salud/MT/Paginas/medicamentos-regulacion-precios.aspx		
	Procurement prices	*Ministerio de Salud y Protección Social*	SISMED (Database) https:// www.sispro.gov.co/central-prestadores-de-servicios/Pages/SISMED-Sistema-de-Informacion-de-Precios-de-Medicamentos.aspx	Open data	Price registry (average price (3 months), minimum price and maximum sale price); maximum selling price, minimum selling price, quantity and number of contracts
	Reimbursement policies	Ministerio de Salud y Protección Social	Legislation	Restricted data	Reimbursement of selected high pricing medicines due price caps on inpatient drugs reimbursements by pharmaceutical active ingredient
	Patent information	*Superintendencia de Industria y Comercio*	https://www.sic.gov.co/patentes	Open data	IP report status of each patent (granted, denied, abandoned, expired)
			https://sipi.sic.gov.co/sipi/Extra/IP/PT/Qbe.aspx?sid=637629868357104486		
	Clinical trial Information	Not available	Not available	Not available	Not available
	Information on R&D expenditures	Not available	Not available	Not available	Not available
	HTA	*Instituto de Evaluación Tecnológica en Salud* (IETS)	Decisions can be found on the IETS website: https://www.iets.org.co/Busqueda/FrmResumen.aspx?valor=Apreciaci%C3%B3n%20cr%C3%Adtica	Open data	A report on cost effectiveness, safety, and efficacy and diagnostic validity of health technology and budget impact analysis; clinical practical guidelines. No formal dossier with the recommendation

^a^ Adapted from WHO Europe, 2021 (https://apps.who.int/iris/bitstream/handle/10665/342474/9789289055789-eng.pdf) and https://www.fosteropenscience.eu/taxonomy/term/112

Glossary Adapted from O´Neill et al. (2017) (https://www.fosteropenscience.eu/taxonomy/term/112).

Open access: online, free-of-cost access with limited copyright and licensing restrictions. 1) open access journals, 2) open access repositories, or 3) open archives.

Open data: online, free-of-cost, accessible data that can be used, reused, and distributed provided that the data source is attributed and shared alike (private sector, UN, or NGO). Data not protected by rule, therefore public. No prior authorization by the data manager is required and will be carried out through existing channels for open data and active transparency.

Open government data: online government data offered free-of-cost, with terms that allow its reuse and redistribution.

Open data use and reuse: online and free-of-cost data, with terms that allow its reuse and redistribution.

Restricted access: sharing of journals, repositories, or archives subject to the permission of external access by the information manager.

Restricted data: data sharing conditioned on the permission of external access by the data manager.

Volunteer initiative: initiative carried out because by choice and not force, without legal obligation, compulsion, or persuasion.

Private initiative: initiative through the private sector.

Legislation: national legislation supporting the transparency of pharmaceutical products.

Type of price data is related to the Glossary of Pharmaceutical Terms updated in 2019 (https://ppri.goeg.at/about_translations).

## Results

### Marketing authorization

In terms of the regulatory market for medicines, the National Administration of Drugs, Foods and Medical Devices (ANMAT) regulates health products and services in Argentina. In Brazil, the Brazilian Health Surveillance Agency (Anvisa) executed the sanitary control of products and services subject to health regulation. Anvisa is an autarchy linked to the Ministry of Health, which coordinates the Brazilian Health Regulatory System (SNVS). In Colombia, the National Institute of Food and Medicines Surveillance (INVIMA) is an autonomous entity attached to the Ministry and implements sanitary surveillance for medicines and other supplies that may impact individual and collective health.

No data on the number of medicines with marketing authorization in Argentina were found. The Anvisa web portal showed 18,888 pharmaceutical market presentations registered in Brazil in 2019, which corresponds to 5897 medicines. Colombia showed 75,881 registered pharmaceutical market presentations in 2018, which corresponded to 534 medicines (Ministerio de Salud y Protección Social, 2017).

In Argentina, Brazil, and Colombia, it is possible to determine the status of review and marketing authorization, Medicine Public Assessment Reports for approval or denial decisions, and leaflets. Assessment of the marketing authorization of medicines in Argentina showed no access to technical advice, including registration or information. Only Brazil provided complete approval or denial reports. Brazil also provides information on changes in protocols, legislation, manuals, and guidelines.

Brazil and Colombia present Active Pharmaceutical Ingredient (API) manufacturers’ data. A complete dossier with all the documentation provided by the industry is not available. The dossiers contain no data on R&D costs or clinical trials. An important gap in the analysis of records is that it is not possible to verify information from previous versions of the record, which hinders the analysis of the reasons for reassessment or analysis of the studies that supported the original record. The inclusion of a new indication in the package insert of the reference product automatically generates inclusion in the package inserts of the respective generic products, thus affecting the production of generic products and creating so-called “second-use patents”.

The transparency aspects related to the marketing authorization of infliximab are shown in Table 1.

#### Warnings and recall

Argentina publishes warnings and recalls related to the lack of availability of medicines. Brazil publishes a database for warnings and recalls on the Anvisa website and in the Official Gazette. In Colombia, recalls and alerts have had a critical moment due to the website not being available during 2022: the INVIMA website does not currently allow access to core information on medicines.

### Pricing

#### Pricing regulation

Regarding medicine pricing, Argentina has no formal legislation for pricing and does not use external or international reference pricing. Nonetheless, the Ministry of Health, National Commission of Health Technology Assessment (CONETEC); Institute for Clinical Effectiveness and Health Policy (IECS), and the Coordinating Unit for Evaluation and Performance of Health Technologies (UCEETS) are involved in adjusting prices in consultation with the manufacturer depending on the market demand. The *Convenio Precio Abonado por el Público* (Agreement on Subsidized Prices for the Public) reduces the prices patients pay for certain therapies.

In Brazil, the maximum selling prices to the public or private sectors are available on a portal, which includes information on rates and discounts. Documents are sent to Anvisa for price analysis through the Sammed information system; however, access to this information is restricted. In 2021, Public Consultation No. 2/2021 was submitted to society, with a proposal for a new Resolution that establishes criteria for setting prices for new products and new medicines presentations. A report with contributions to the proposal or a new Resolution has not yet been published. The National Health Council, through Recommendation no. 030, passed on September 22, 2021, called for the suspension of public consultation for medicine pricing and a broad debate based on the Regulatory Impact Analysis regarding medicine pricing in Brazil. CNS Recommendation No. 054, passed on August 20, 2020, confirmed the role of CMED, especially during the COVID-19 pandemic, and reinforces the need for transparency on production costs and medication logistics.

The Colombian government allows for free pricing by pharmaceutical companies; however, in some exceptional circumstances, the *Comisión Nacional de Precios de Medicamentos y Dispositivos Médicos*—CNPMDM can put a medicine under direct control, including public health interest or high financial burden, unjustified price increases, prices above the inflation levels recorded for the previous year, and when the national reference price for a relevant market is significantly above its international reference price. The reference price is determined, and the maximum sale price is defined (Ministerio de Salud y Protección Social, 2018). According to Vargas et al., 2022, the prices of 2,513 medicines were capped as of September 2021. In Colombia, prices are negotiated, with a maximum admissible price for public purchases or reimbursement. There is co-payment by users of a variable percentage of the sale price of medicines and parallel imports.

#### Procurement prices

Argentina lacks price regulation but provides price negotiation at the national level. The market price is registered voluntarily by suppliers; thus, the information is biased. Moreover, the unit value or quantity purchased is not provided. The public purchase bids for medicines can be obtained. The manufacturer-suggested prices are collected as the final price to the public from surveys of the pharmaceutical industry. All medicines included in the Compulsory Medical Plan (*Programa Medico Obligatorio*—PMO) are reimbursed by the government. The costs of prescription drugs are not publicly available. In 2021, there was a value-based agreement in operation (for bevacizumab) in Argentina; however, there are no published details on this contract due to commercial confidence.

In Brazil, information and data on public purchases of medicines can be accessed through the Health Price Database (BPS), which includes item data, purchase data, manufacturer/supplier data, institution/location data, Maximum Government Procurement Price (*Preço Maximo de Venda ao Governo*—PMVG), highest purchased price, lowest purchased price relative to the lowest unit supplied, and also through the Ministry of Economy Price Panel, which provides information on the number of purchase contracts and average, median, highest, and lowest prices per supply unit. Any discounts on the final price are not published. Brazil also provides information on public procurement contracts for medicines, including bid waiver extracts, auction notices, and additive term extracts. Only the global amount of installment is available; individual amounts are not available, nor is the amount available from the Official Gazette website. The Transparency Portal provides details of the contract, value, and quantity but does not include information on discounts on the final price. Regarding reimbursement policies, per capita funding is provided for certain essential medicines and medicines used in the prison system. Information on purchase agreements with public laboratories and purchases through international organizations like Pan American Health Organization (PAHO) is opaque. Reimbursement is also made to pharmacies included in the *Programa Farmácia Popular* and payment for medications linked to hospital procedures. Only the overall value of the installment from federal purchasing is available; contract details and individual values or quantities are not available for high-price medicines. Discounts or taxes on the final price are not available.

Since 2006, an important achievement to promote access to medicines in Colombia has been pricing regulation, which monitors the pharmaceutical market based on The Medicine Price Information System (*Sistema de Información de Precios de Medicamentos*—SISMED), a public data source where market actors periodically report commercial transactions and contains historical information on sales volumes and medicine prices and is an important auxiliary tool for the price regulation mechanism in Colombia. It provides quarterly reports on the national volume of medicines and expenditures from wholesalers, the pharmaceutical industry, and healthcare providers. Since 2007, the SISMED cube has allowed anonymous access to the report’s microdata as an additional mechanism for the analysis of this information. No public databases exist to trace medicines or their availability in the three countries (Table 2).

### Intellectual property

A pharmaceutical product may be awarded patents on different sub-components and not only on the substance but also the production process. Regarding intellectual property, in Argentina, the National Institute of Industrial Property (INPI) provides a database that includes denied patents. Argentina provides an IP report status for each patent (submitted -in process-, abandoned, denied, forced withdrawal, granted, or expired). In Brazil, patent applications are analyzed in the database provided by the National Institute of Industrial Property (INPI). Free registration is required to gain access to its documents. Law 14,195, published on August 27, 2021, established the end of prior consent for pharmaceutical patents by Anvisa. In Brazil, the INPI website provides a database with technical information on patent applications and registrations in the country, although it is not very user-friendly. This system provides a summary, claim, descriptive report, and any drawings of the patent application. Information not published in the database can be requested by email (copdocpat@inpi.gov.br). Access to some documents may be sent by companies in response to requests for data/information from the INPI; however, it is not possible to access documents issued by the INPI. The INPI also publishes the Intellectual Property Journal *(Revista de Propriedade Intelectual*) monthly, which contains all its acts, orders, and decisions relating to the industrial property system in Brazil. Colombia provides the IP report status of each patent (granted, denied, abandoned, or expired).

Patents with pending status can generate insecurity in the production of generics of the linked drug and, consequently, generate a monopoly on a patent that was not even granted.

### Clinical trials

None of the three counties has an official public online database with timely information on preclinical studies (data and methodology), pharmacology, and specific toxicology. Neither Argentina nor Brazil includes clinical trial IDs, while Brazil includes the protocol ID of the sponsor.

Regarding clinical trials: Argentina provides databases on clinical trials and publishes a list of denied or disinvested studies (Table 3). In Brazil, as of RDC 36/2012, clinical trials that require Anvisa consent must now prove the registration or submission of clinical trials in the Brazilian Registry of Clinical Trials (ReBEC) or the International Clinical Trials Registration Platform (ICTRP/WHO) databases, which contribute to the transparency of clinical research data. Through Anvisa, Brazil also makes available the panel consultation of authorized clinical trials.

**TABLE 3:** Intellectual Property and Health Technology Assessment health care decision.

Attribute	Medicines	Argentina	Brazil	Colombia
Intellectual Property	Aflibercept	2 granted patent, forced to withdraw	6 patent registrations	1 request denied
	Etanercept	7 granted patent, forced to withdraw and 1 patent denied	10 patent registrations, 1 technology transfer request, 9 pending requisition; and 6 applications rejected, either archived, or expired	6 denied requests
	Infliximab	—	3 patent registrations	2 applications under review
	Rituximab	—	14 patent registrations, 1 technology transfer request, 14 pending requisition; and 29 applications rejected or filed or expired	6 patent registrations, of which 2 granted, 2 denied, one abandoned, and one under the figure of “publicada sin pago”
	Tocilizumab	—	3 patent registrations, 5 pending requisition; and 3 applications rejected, either archived or expired	1 application in expired status
	Trastuzumab	2 granted patent, forced to withdraw and 1 abandoned patent	8 patent applications, 1 technology transfer request, 21 pending requisition; and 9 applications rejected or failed or expired	1 patent granted, expires July 2023
Health Technology Assessment healthcare decision	Aflibercept	—	Aflibercept has 2 technical reports recommending incorporation into SUS. Incorporation ordinance conditions the incorporation of aflibercept for the treatment of patients with diabetic macular edema, conditioned to price negotiation based on the proposal presented by the applicant and the elaboration of the Clinical Protocol and Therapeutic Guidelines of the Ministry of Health	—
			1- Aflibercept for diabetic macular edema. Decision: incorporate to SUS. 06 November 2019	
			2- Aflibercept and ranibizumab for the treatment of neovascular age-related macular degeneration (AMD) in patients over 60 years of age. Decision: Incorporate into SUS. 10 May 2021	
	Etanercept	—	Etanercept has 1 technical report recommending no incorporation and 2 technical reports for incorporation. incorporation ordinance which includes the reduction of treatment cost as a condition for acquisition	—
			Etanercept in the first stage of psoriasis treatment after first-line failure in pediatric patients. Decision: Incorporate into SUS. 31 October 2018	
			1- Biological medicines for the treatment of rheumatoid arthritis. Decision: Incorporate into SUS. 11 September 2012	
			2- Biological medicines for the treatment of moderate to severe psoriasis in adults. Decision: Not to be incorporated into the SUS. 05 October 2012	
	Infliximab	—	Infliximab has 3 non-incorporation reports and 2 incorporation reports and signs of treatment cost reduction as a condition for acquisition	—
			1- Biological medicines for the treatment of rheumatoid arthritis. Decision: Incorporate into SUS. 11 September 2012	
			2- Biological medicines for the treatment of moderate to severe psoriasis in adults. Decision: not to incorporate into the SUS. 05 October 2012	
			3- Infliximab for severe ulcerative colitis. Decision: not to incorporate into the SUS. 07 July 2014	
			4- Infliximab for the treatment of psoriasis. Decision: not to incorporate into the SUS. 31 October 2018	
			5- Infliximab, adalimumab, golimumab, and vedolizumab for the treatment of moderate to severe ulcerative colitis. Decision: Incorporate into SUS. 23 October 2019	
	Rituximab	—	Rituximab has 3 non-recommendation technical reports and 2 SUS incorporation recommendation reports	—
			1- Subcutaneous rituximab for the treatment of previously untreated CD20-positive B-cell follicular non-Hodgkin’s lymphoma in combination with chemotherapy. Decision: not to incorporate into the SUS. 11 July 2018	
			2- Subcutaneous rituximab for the treatment of CD20-positive, diffuse large B-cell non-Hodgkin’s lymphoma in combination with chemotherapy. Decision: not to incorporate into the SUS. 11 July 2018	
			3- Rituximab - Biological medicines for the treatment of rheumatoid arthritis. Decision: incorporate to SUS. 11 September 2012	
			4- Rituximab for the treatment of CD20-positive, B-cell, follicular, non-Hodgkin’s lymphoma. Decision: not to incorporate into the SUS. 26 August 2013	
			5- Rituximab for the treatment of 1st and 2nd line, cd20 positive, b-cell, follicular, non-Hodgkin’s lymphoma. Decision: incorporate to SUS. 30 December 2013	
	Tocilizumab	COVID Health Technology Assessment Report No. 06. Tocilizumab for the treatment of patients with COVID-19, Final report. 21 May 2021. https://www.argentina.gob.ar/salud/conetec/actualizaciones. The Reports are previously submitted to Public Consultation. https://www.argentina.gob.ar/salud/conetec/informes-de-ets	Trastuzumab has 2 technical reports recommending no incorporation and 1 technical report for incorporation. Incorporation Ordinance which includes the reduction of treatment cost as a condition for acquisition	Inform. Analysis of the presumptive impact of adalimumab, infliximab, tocilizumab, abatacept, certolizumab pegol, and golimumab in patients with rheumatoid arthritis refractory to treatment with non-biological disease-modifying anti-rheumatic medicines. 01 October 2013. https://www.iets.org.co/Archivos/Informe%20AIP%20Artritis%20VF.pdf Other technical reports: https://www.iets.org.co/documentos-tecnicos/
			1- Tocilizumab monotherapy in the second stage of treatment of moderate to severe rheumatoid arthritis. Decision: Not to be incorporated into the SUS. 22 September 2016	
			2- Tocilizumab for the treatment of rheumatoid arthritis - 1st line treatment with biologics after failure of synthetic DMARDs. Decision: Not to be incorporated into the SUS. 11 September 2012	
			3- Biological medicine for the treatment of rheumatoid arthritis. Decision: Incorporate into SUS. 17 March 2015	
	Trastuzumab	Health Technology Assessment Report nº14. Subcutaneous trastuzumab in the treatment of HER2-positive breast cancer. Final report. March 2021. https://www.argentina.gob.ar/sites/default/files/informe-14-trastuzumab_0.pdf	Trastuzumab has 3 incorporation reports and respective incorporation ordinances, which include treatment cost reduction, molecular examination requirement, and monitoring of clinical results as a condition for acquisition	—
			1- Trastuzumab for the treatment of first-line metastatic HER2-positive breast cancer. Decision: incorporate to SUS. 03 August/2017	
			2- Trastuzumab for the treatment of advanced breast cancer. Decision: incorporate to SUS. 26 July 2012	
			3- Trastuzumab for the treatment of early breast cancer. Decision: Incorporate into SUS. 26 July 2012	

Sources:

IP https://portaltramites.inpi.gob.ar/PatenteConsultas/BusquedaParametros; https://busca.inpi.gov.br/pePI/servlet/LoginController?action=login; https://sipi.sic.gov.co/sipi/Extra/IP/PT/Qbe.aspx?sid=637629868357104486 and https://ipportal.wipo.int/.

HTA https://www.argentina.gob.ar/salud/conetec/informes-de-ets; https://www.gov.br/conitec/pt-br/assuntos/avaliacao-de-tecnologias-em-saude/recomendacoes-da-conitec and https://www.iets.org.co/documentos-tecnicos/

^a^ BRASIL, Biological Medicines (infliximab, etanercept, adalimumab, rituximab, abatacept, tocilizumab, golimumab, and certolizumab pegol) for the treatment of rheumatoid arthritis, considering that the incorporation of all biological alternatives available on the market for the treatment of rheumatoid arthritis will occur exclusively if treatment costs are reduced. All incorporated medicines must follow a Clinical Protocol and Therapeutic Guidelines (PCDT) before being made available. The deadline for availability in the SUS is up to 180 days after incorporation.

Colombia grants 5 years of test data exclusivity for new medicine (medicines with chemical entities not yet registered for commercialization). Decree 2085 of 2002 describes two requirements for granting exclusivity: a new chemical entity and considerable effort. The Decree excludes new uses and new formulations, exclusivity for pediatric use, orphan drugs, new indications, and new routes of administration/formulations.

The form of action that institutions can take regarding what is regulated, including technical advice about the denial of registration. Therefore, transparency can be implemented through institutional agreement and deliberation, without the need for legal determination. Table 3 shows the transparency attributes categorized as related institutions, data accessibility taxonomy and information, and type of data/information.

### Research and development

No official public online databases are available with information on R&D expenditures for products with marketing authorization in Argentina, Brazil, or Colombia.

Furthermore, no official public online databases are available with timely information on specific preclinical studies (data and methodology), pharmacology, and toxicology.

Regarding R&D, Brazil provides general information on pre-clinical research projects and the opinion of the ethics committee through Plataforma + Brasil.

### Health technology assessment

In April 2018, the government of Argentina created the CONETEC, which will be responsible for evaluating new medicines, medical devices, and clinical procedures and generating information on the cost-utility and cost-effectiveness of health technologies, as well as the budget impacts on economic and social conditions in the country. HTA evaluations will be used to determine whether new medicines will be included or excluded from the list of reimbursed technologies. The main legal bases related to this theme are Resolution 623/2018, which creates the National Commission for the Evaluation of Health Technologies; the Operational Manual of Structure and Operation—Technical Document nº 1—CONETEC and Resolution 153/2019, which addresses the Procedure for Submitting Proposals for Incorporations, new Indications and/or Exclusions from the Health Technology Registry subject to Reimbursement. Resolution 370-E/2017 deals with information for MAH of Health Technologies on the Mandatory Medical Program. The manufacturer applies to the Superintendencia de Servicios de Salud (SSSalud) to post their medicines into the Programa Medico Obligatorio, which establishes the essential basic benefits that Social Works and Insurance Agents must guarantee to the entire beneficiary population and includes lists of medicines that appear in Annex III grouped according to their various doses and pharmaceutical forms. The reference price is established, 40% of which represents minimum mandatory coverage for Social Works and Health Insurance Agents.

In Brazil, technologies are evaluated by the National Commission for the Incorporation of Technologies in the Unified Health System (Conitec), which advises the Ministry of Health (MoH) in the process of incorporating, excluding, or changing pharmaceuticals offered under the Brazilian National Health System (SUS). After the recommendation made by Conitec, the MoH will report the final recommendation by the Secretary of Science, Technology, Innovation, and Strategic Inputs of the MoH. After that, the committee formed by representatives of the federal government, states, and municipalities will decide how the financing of the product will be incorporated into the SUS; that is, which government instance will acquire and pay for the product. For incorporation into the SUS, HTA studies are carried out regarding scientific evidence (effectiveness, safety, and effectiveness) and economic assessment (cost-effectiveness). HTAs act as a tool for the efficient use of health resources and to promote access to more effective technologies to meet the real needs of patients. For some technologies incorporated into the SUS, it is a sign of reduced treatment cost as a condition for public acquisition. In the case of rare diseases, it is increasingly essential to assess the opportunity cost and the impacts of high prices in a health system with limited resources. While the effectiveness of technologies for rare diseases must be evaluated through information systems and real-world studies, given the disruptive nature of these technologies and the innovations that have been increasingly present, measures such as risk-sharing agreements can also be potent alternatives. Law n°. 14,307, passed on March 3, 2022, introduced the use of HTA and the predictability of deadlines in the process of updating coverage in the field of private health and instituted the Commission for Updating the List of Procedures and Events in Supplementary Health. Law nº. 14,313, passed on March 21, 2022, determines that the methodologies, indicators, and cost-effectiveness parameters used in the economic evaluation in analyzing the incorporation of technologies into the SUS by Conitec be regulated and widely disseminated.

Although it was already foreseen in the regulations created by Conitec in 2012, it was only in October 2021 that the reassessment of technologies incorporated into the SUS began, which had as a constraint its reassessment after 3 years of use. As of May 2022, three technologies have been re-evaluated (Brazil, 2022). These reassessments include updating the scientific evidence on efficacy, effectiveness, and safety; analysis of data on the use of technology in the SUS (real-world data); comparisons of the expected budgetary impact of incorporation after implementation in the SUS, update of the monitoring of the technological horizon and patent protection of the technology (Brazil, 2022). Conitec also publishes studies to evaluate the performance of technologies incorporated in the SUS and reports or alerts with Horizon Scanning on new and emerging health technologies (Brazil, 2022).

Colombia leverages HTAs for inclusion in the national formulary. The *Instituto de Evaluación Tecnológica en Salud* (IETS) is the main entity performing HTAs in Colombia. In December 2018, Decree 433 was implemented, which allowed higher prices for medicines offering better results than comparable medicines currently on the market and limiting prices for medicines with inferior results. The national formulary is updated every 2 years when the MoH asks the IETS to evaluate the effectiveness, safety, and budget impact of diseases of interest. Separately, the government can reimburse the medicine on a case-by-case basis if providers can justify its use. IETS publishes a report on the cost-effectiveness, safety, efficacy, and diagnostic validity of health technologies as well as clinical practical guidelines. No formal dossiers with such recommendations are available.

### Characterization of data on selected medicines

#### Characterization of health technology assessment data for selected medicines

In Argentina, only two reports have been published (both in 2021): subcutaneous trastuzumab in the treatment of HER2-positive breast cancer and tocilizumab for the treatment of patients with COVID-19. This HTA process is recent in Argentina.

In Brazil, the recipients of the incorporation of possible medicines or use were identified. The Conitec decision was also identified as the condition for public purchase and possible report with medicines and access to the video recorded. Payment or registration to access the information portal was not required.

In Colombia, only the budget impact related to these selected medicines (infliximab, trastuzumab, rituximab, tocilizumab, aflibercept, and etanercept) was found.

#### Characterization of public procurement data for selected medicines

To promote transparency in public procurement, in general, the databases analyzed belonged to governmental entities such as ministries or public procurement purchasing centers and private entities. The data sources in Argentina included K@iros^®^ and *Vademecum Nacional de Medicamentos* (VNM). The latter provides public information and data restricted to registered users. Both portals report the last Pharmacy Retail Price (PRP). The *Vademecum Nacional de Medicamentos* (VNM) includes the certificate number, laboratory, trade name, pharmaceutical form, presentation, retail price, generic, availability (but no information), GTIN, sale condition, and route of administration. The K@iros^®^ includes publication date, laboratory, pharmaceutical form, presentation, price for sale to the public, and generic information. The sales prices are the same in both data sources.

Additionally, in Brazil, we identified data sources from the BPS and the Ministry of Planning Price Panel. In both systems, the number of purchases and the average and median times between purchases can be identified. Furthermore, in addition to including the supplier, the systems provide the purchasing institution (military, federal, subnational health services, and hospitals). Despite the two databases including the same information, the prices differed.

Finally, in Colombia, SISMED includes the number of registered purchases and the maximum and minimum unit prices. This data source is updated quarterly. The data sources in Brazil and Colombia build price indices. In Colombia, the price index corresponds to a quarterly weighted average price of all national reports for each commercial brand, including the various commercial presentations. The medicine coding in the data sources is not uniform (Table 4).

**TABLE 4:** Procurement prices.

Attribute	Medicines	Argentina	Brazil	Colombia
Procurement prices	Aflibercept	No data	BPS 25 mg/ml SANOFI MEDLEY 1.127,47 2.254,9 BAYER 3.200,00 40 mg/ml BAYER 2.518,29 2.916,66 3.132,28 3.170,47 3.348,50 3.348,50 Panel of prices of the Ministry of Economy 25 mg/ml SOLIDOR R$ 27 R$ 28 ZALTRAP R$ 1436,82 BAYER EYLIA R$ 2612,5 R$ 2750 R$ 3800 40 mg/ml BAYER EYLIA R$ 2750 R$ 2900 R$ 3050 R$ 3068,08 R$ 3132,28 R$ 3170,478 R$ 3192,35 R$ 3192,37 R$ 3265,9 R$ 3278,38 R$ 3290 R$ 3294,72 R$ 3318,03 R$ 3344,96 R$ 3344,96 R$ 3385,74 R$ 3385,75 R$ 3598 R$ 3706,45	Number of records: 5 Minimum unit price: $2′671.979 COP Maximum unit price: $2′918.431 COP
	Etanercept	No data	BPS 25 mg/ml PFIZER R$ 679,22 R$ 2.505,41 WYETH R$ 770,33 50 mg/ml PFIZER R$ 1.502,71R$ 1.502,71 SANDOZ R$ 685,54 R$ 750,00 R$ 930,30 Painel de preços Ministério do Economia 25 mg/ml EMBREL R$ 139,41 R$ 626,35 R$ 771,36 R$ 798,2 R$ 798,2 50 mg/ml EMBREL R$ 160,75 R$ 284,39 R$ 500 R$ 523,00 R$542,28 R$ 550 R$ 553,64 R$ 553,65 R$ 615 R$ 638,59 R$ 673,1925 R$ 1320,01 R$ 1493	Number of records: 15 ETANAR 25 mg Minimum unit price: $200.340 COP Maximum unit price: $401.910 COP ENBREL 25 mg Minimum unit price: $252.810 COP Maximum unit price: $388.763 COP ENBREL 50 mg Minimum unit price: $555.041 COP Maximum unit price: $2′220.164 COP
	Infliximab	Vademecum Nacional de Medicamentos (VNM) AVSOLA. AMGEN $104.366.77 IXIFI. PFIZER $107.636,47 REMICADE. JANSSEN CILAG. $114.825,85 REMSIMA. GOBBI-NOVAG. $133.412,50 Kairos web AVSOLA. AMGEN 01/10/2021. $104366.86 IXIFI. PFIZER 09/02/2021 $107636.47 REMICADE. JANSSEN CILAG $114825.85 19/01/2021 REMSIMA. GOBBI-NOVAG 21/10/2021 $133412.50	BPS REMSIMA R$ 1.345,3300 and R$ 1.731,2600 XILFYA R$ 1.731,2600 and R$ 2.583,8800 REMICADE R$ 3.065,6200 Panel of prices of the Ministry of Economy BIONOVIS R$ 773,38 and R$ 776,36 WYETH R$ 800,00 and R$ 1400 XILFYA R$ 851,00 and R$ 1.000,00 REMSIMA R$ 1050 R$ 1169 R$ 1250 R$ 1300 R$ 1330: R$ 1400 R$ 1450 R$ 1485: R$ 1501 R$ 1600 REMICADE 100 MG R$ 2647,7 R$ 2664,66	Number of records: 7 REMSIMA Minimum unit price: $615.300 COP Maximum unit price: $1.483.004 COP REMICADE 100 mg Minimum unit price: $720.000 COP Maximum unit price: $1′483.624 COP
	Rituximab	No data	BPS 10 mg/ml SANDOZ R$ 271,78 R$ 312,99 R$ 441,8267 R$ 1.035,86 R$ 1.433,00 TRUXIMA R$ 375,70 R$ 1.449,00 R$ 1.540,00 R$ 2.000,00 R$ 2.140,00 R$ 2.200,00 WYETH R$ 3.329,65 ROCHE R$ 5.350,00 R$ 5.909,38 Panel of prices of the Ministry of Economy 10 mg/ml TRUXIMA R$ 264,99 R$ 280 R$ 284,13 R$ 288 R$ 292 R$ 292,52 R$ 293 R$ 296,52 R$ 299,34 R$ 300 R$ 303,47 R$ 308,67 R$ 318 R$ 340 R$ 360 R$ 370 R$ 380 R$ 390: R$ 400 R$ 436 R$ 469,88 R$ 485,19 R$ 530 R$ 600 R$ 999,99 R$ 1400 R$ 1420 R$ 1448,46 R$ 1455,3 R$ 1464 R$ 1477 R$ 1486 Sandoz BIONOVIS R$ 303,35 R$ 309,14 R$ 312,50 R$ 312,84 R$ 326,45 RIXIMYO R$ 321,58 R$ 331,35 R$ 385 R$ 1370 R$ 1482,64 ROCHE/MABTHERA R$ 1263,43 R$ 1337	Number of records: 22 MABAL 500 mg Minimum unit price: $4′227.383 COP Maximum unit price: $4′227.383 COP MABAL 100 mg Minimum unit price: $845.476 COP Maximum unit price: $1′690.953COP MABTHERA 100 mg Minimum unit price: $845.476 COP Maximum unit price: $3′496.268 COP MABTHERA 500 mg Minimum unit price: $2′331.106 COP Maximum unit price: $4′227.382 COP MABTHERA 1400 mg Minimum unit price: $3′088.892 COP Maximum unit price: $6′598.325 COP RIXATHON 100 mg Minimum unit price: $874.068 COP Maximum unit price: $4′227.382 COP TRUXIMA Minimum unit price: $845.477 COP Maximum unit price: $4′295.782 COP
	Tocilizumab	No data	BPS 20 mg/ml ACTEMRA R$ 436,34 R$ 568,25 180 mg/ml ACTEMRA R$ 954,55 Panel of prices of the Ministry of Economy 20 mg/ml ACTEMRA R$ 164,75 R$ 436,34 R$ 436,34 R$ 465,96 R$ 568,25 R$ 1420,66 R$ 1456,19 R$ 1737,74 R$ 1776,89 R$ 1810,46 R$ 1855,73 180 mg/ml ACTEMRA R$ 893,86 R$ 1.216,4625 R$ 1655,06 R$ 1799,49	Number of records: 3 ACTEMRA 200 mg Minimum unit price: $734.808 COP Maximum unit price: $1′016.486 COP ACTEMRA 80 mg Minimum unit price: $293.924COP Maximum unit price: $353.468 COP ACTEMRA 162 mg Minimum unit price: $571.387 COP Maximum unit price: $2′675.776 COP
	Trastuzumab	Vademecum Nacional de Medicamentos (VNM) HERCEPTIN. ROCHE. $355.331,95 HERCEPTIN SC. ROCHE. $355.331,95 KADCYLA. ROCHE. 100 mg Vial x 1 29/10/2021 $412.640,43 y 160 mg Vial x 1 29/10/2021 $660.224,74 PERJETA-HERCEPTIN COMBO PACK. ROCHE. $855.700,48 KANJINTI. AMGEN. $238.993,37 TRAZIMERA. PFIZER. $249.896,55 TUZEPTA. RAFFO. $278.638,38 Kairos web HERCEPTIN. ROCHE. 29/10/2021 $355331.95 HERCEPTIN SC. ROCHE. 29/10/2021 $355331.95 KADCYLA. ROCHE. 100 mg Vial x 1 29/10/2021 $412640.43 y 160 mg Vial x 1 29/10/2021 $660224.74 KANJINTI. AMGEN. 01/10/2021. $238993.37 TRAZIMERA. PFIZER. 28/10/2021. $249896.60 TUZEPTA. RAFFO. 26/10/2021. $278638.43	BPS 440 mg lyophilized powder for solution for injection HERZUMA R$ 3.600,00 CMED R$ 7.165,55 120 mg/ml solution for injection HERCEPTIN R$ 8.487,3900 and 8.487,3900 Panel of prices of the Ministry of Economy 440 mg lyophilized powder for solution for injection 20 compras ZEDORA R$ 2090 R$ 2391,31 R$ 2398 R$ 2650 HERZUMA R$ 2200 R$ 3100 R$ 3200 R$ 3544,84 R$ 3600 R$ 3900: R$ 4610,7 R$ 5502,16 TRAZIMERA R$ 2300 R$ 2700,05 R$ 4243,27 R$ 4395 R$ 4460,03 HERCEPTIN R$ 10009,05 R$ 10016,71 120 mg/ml solution for injection HERCEPTIN R$ 9902,42 R$ 10194,13 R$ 10849,06	Number of records: 15 OGIVRI Minimum unit price: $2′855.700 COP Maximum unit price: $5′372.695 COP TRAZIMERA Minimum unit price: $2′685.120 COP Maximum unit price: $5′372.695 COP HERCEPTIN 440 mg Minimum unit price: $5′372.695 COP Maximum unit price: $5′372.695 COP HERCEPTIN 600 mg Minimum unit price: $ 2′868.150 COP Maximum unit price: $6′355.960 COP HERCEPTIN 150 mg Minimum unit price: $ 1′213.867 COP Maximum unit price: $1′831.600 COP

#### Characterization of intellectual property data for selected medicines

In terms of transparency, according to the “national transparency check-list on medicines and health products” developed in August 2019 by the *Observatoire de la transparence dans les politiques du medicaments*, a patent registry website is expected to provide full access to the full documents of the patent examiners’ analysis and decisions; up-to-date information about each step of the examination process, and information on technology transfer agreements related to the patent or application, voluntary licenses related to the patent or application, and compulsory license or government use issued for the patent.

It was possible to identify the patent applications for each of the products in this country in all three countries, including requests for molecules and processes. We also identified which had technology transfer requests. However, it was difficult to identify the duration of the patent and whether the type of patent guaranteed a monopoly of the innovation or an extension of the monopoly term (Table 4).

## Discussion

The results of this study provide evidence of the limitations of the infostructures (organizational capacity for collecting and distributing information) related to the pharmaceutical value chain in the three countries for appropriate measures to publicly share data and information on pre-clinical and clinical findings and costs. None of the three countries showed enhanced availability and access to aggregated results data and, if already publicly-available or voluntarily-provided, costs from human subject R&D, clinical trials, or the respective underlying assumptions for the calculations. However, the correlation between the capacity for innovation in R&D in peripheral countries and the demand to delink R&D costs and clinical trials must be considered, as the pharmaceutical industry in the region mainly produces generic medicines and has not yet been able to meet national or regional needs.

Regarding pricing information, the three countries do not provide detailed and standardized prices, rates, discounts, and quantities related to price negotiations, contracts, and payments for each medicine purchased, tax legislation, the volume of purchases, and the time between acquisitions. The pricing collection method varies across these countries. These findings corroborate the findings reported by Chaves et al. (2021), who mentioned the lack of clarity in the information made available by Brazil on the prices charged on the public market. The information found for the countries in the present study was not related to the exact data of purchases of medicines, regulatory purposes for medicine’s prices, or price catalogs reported by surveys of manufacturers or retailers. This lack of harmonization makes it challenging to draw firm conclusions related to comparative analyses of pharmaceutical products. Not all primary arrangements are publicly reported in a timely fashion. Those that are reported often lack basic information such as the total number of technologies provided and their prices. The related terms and conditions (such as timelines for delivery) are also often unavailable. Information on how the total price and/or unit price were determined or what is included in the reported values is rarely reported. Some countries may include shipping and handling costs or investments in manufacturing in their reported total prices, while others do not. The Portal Nacional de Contratações Públicas (PNCP) (*National Public Contracting Portal*) is being implemented in Brazil, whereby mandatory information related to the public procurement process must be systematized and published according to Law No. 14,133/2021.

The secrecy of pharmaceutical pricing and umbrella or framework procurement agreements further weakens traceability, making it difficult to know the unit price paid by the government. Due goods, lots, or services can be purchased throughout the period of the agreement; however, costs may change due to contract modification and payment and delivery schedules may differ (Fazekas et al., 2021). The World Bank performed an analysis using public procurement data gathered by governments’ electronic procurement systems in seven countries and two territories across Latin America. They obtained the data sets directly from the official data holders such as national public procurement authorities and made product classification, harmonization, unit prices, contract values, and procurement methods (Fazekas et al., 2021).

Another challenge is the opacity of the prices paid for medicines linked to general procedures, such as surgery or cancer treatments. The WHO technical report on the pricing of cancer medicines and its impact noted that pharmaceutical companies “chose not to launch or delayed the launch of medicines in countries with lower capacity to pay, irrespective of whether prices were disclosed or not” (WHO, 2018a). Moreover, according to the WHO Europe report, these companies “have knowledge about the discounts and rebates offered to all purchasers, giving the pharmaceutical industry an advantage in negotiation”, which can lead to discrepancies in price comparisons (WHO Regional Office for Europe, 2021).

This issue harms global, regional, and subnational analyses and comparisons and can facilitate corruption and exploitative pricing, and increase the negotiating power of the pharmaceutical industry and health system inefficiency. Moreover, there is no substantial evidence that this posthumous transparency can increase the accountability of the parties involved or reverse any problems identified based on the data and information available. Difficulties in accessing physical documents or large amounts and/or fragmented information can be a limiting factor for drawing quality conclusions. Acosta et al. (2018) recommend “implementing cooperative efforts that reduce the variability of the presented data and avoid bias when comparing information,” “that websites publish documents that describe in detail the components of each price (for example, whether or not VAT is included and, if so, the tax amount),” and “Websites that report public procurement should include variables that can explain or justify price variations (transaction volume, packaging size, payment terms, etc.)”.

Regarding marketing authorization, it is not possible to access the complete documentation presented by the companies. Although Colombia allows data exclusivity, it is not possible to identify the parts of data purported to be subject to data exclusivity. Silva et al. (2016) suggested complete data in clinical trial repositories are important for “more comprehensive analyzes regarding biases, conduct failures or even ethical failures” In May 2022, the World Health Assembly approved a resolution that aimed to improve the coordination, design, and conduct of clinical trials worldwide, and called on countries to ensure that publicly funded trials make their results public on a trial registry within 12 months; however, the resolution did not mention transparency on the costs of clinical trials. These cost data are essential to compare the costs of conducting clinical trials and relate them to the outcomes (Bruckner, 2018).

Pre-clinical and clinical data, the open collaborative research framework shared among actors, can provide a legal framework for the regulation of clinical trials, accelerate the development of new drug combinations, and prevent potential fraud and falsification of clinical data and information by all directly involved parties (hospitals/clinics/health facilities, investigators, sponsors, auditors, and the independent ethics committee) (WHO, 2018b). The UCL Institute for Innovation and Public Purpose (2018) suggested “a collaborative research process with equitable sharing of risks and rewards among actors, and long-term horizons to deliver accessible products.” The ability to test candidate compounds in combination during early-stage medicine development due to such open collaborative frameworks for R&D would accelerate the development of new drug combinations.

Despite the abstract and claims of the patent application along with the current submission status and guidelines for patent examination, no information is available on information sharing and mutual assistance between the patent office and competition authorities in the country. Paranhos et al. (2021) warned that the use of data exclusivity in Brazil, in addition to being unnecessary to attract innovative products, could “further hinder the development of the newly created national market for biosimilars, causing delay in the entry of these medicines into the country, that would find itself dependent on foreign companies.” Global strategies such as open licensing or patent pools must be discussed at the national level, especially in the global south (Krishnan, 2022).

Caetano et al. (2019) suggested that the successful implementation of these strategies to be successful in Brazil would require a transparent HTA, including prioritization criteria; they also stated that “a legal, ethical framework and structural conditions are needed to monitor effectiveness, conditions that Brazil still does not have.” The main challenges to improving the transparent and adequate HTA include the establishment of a registration system and web service for national and subnational data, the identification of research groups trained to carry out prospective/longitudinal studies, the inclusion of data on the purchase of pharmaceutical products in the territories, and the adequacy of the time needed to measure outcomes.

Transparency must be linked with the mandatory equitable distribution of medicines and other technologies such as the mechanism or Pandemic Influenza Preparedness (PIP) Framework, exclusive to influenza viruses or COVAX, which has shown limited success. The Institute of Technology in Immunobiologicals of the Oswaldo Cruz Foundation (Bio-Manguinhos/Fiocruz) in Brazil and the biopharmaceutical company Sinergium Biotech in Argentina were selected for the development and production of mRNA vaccines in Latin America, as a strategy to face pandemics toward the distribution and equitable access of these technologies in the region *via* the PAHO Revolving Fund (Organização Pan-Americana da Saúde, 2021).

### Data access

The main challenges included data heterogeneity (accuracy and format), data fragmentation (multiple databases, websites, and multiple owners/stakeholders), data availability (protection for commercial or related reasons), and lack of data conceptualization (ontologies).

### Capacity to work collaboratively

Working collaboratively to enhance information reporting by all stakeholders can improve national capacities through international cooperation and open and collaborative research across the entire pharmaceutical supply chain. The UCL Institute for Innovation and Public Purpose (2018) suggested that “public funding should support and stipulate participation in open data repositories, open access publishing and collaborative research initiatives.” However, additional discussion is required on the risk for data transparency and evidence in the pharmaceutical chain, instead of strengthening good governance and supporting the rational use of medicines, to become a tool for the consultation of core countries and the use of evidence and data by core countries (including their pharmaceutical industries) to maximize profits and deepen the inequity in access to technologies. The role of each political institution and stakeholder in healthcare policy and the organization of the national healthcare system must be considered to assess the capacity for collaborative work between these countries (Marmor and Wendt, 2012).

This study demonstrated that the lack of standardization of report requirements, quantities and units of measures and information, harmonized terminology, and reporting are important challenges to working collaboratively and collaborative analysis. Due to other national rules, cooperative medicine assessment and price negotiation can be unfeasible, such as the attempt by Mercosur to jointly purchase medicines.

## Conclusion

The results of the present study reinforce the information asymmetry between stakeholders; demonstrate the fragmentation, data gaps and overlaps, and difficulty in comparing available data across the three countries; and underscore the underutilization of research evidence and the need for knowledge translation. Although Brazil showed better information and more transparency, the three countries must advance in strategies to improve the provision of medicine value chain data. Such data should be treated as a public good that is findable, accessible, interoperable, and reusable.

Open data can be used to develop repositories that provide non-trivial knowledge (Knowledge Discovery in Databases, KDD) on the medicine production chain and input for better quality pharmacoeconomic studies. Moreover, although pharmaceutical policy decisions are informed by evidence, transparency is essential as it is oriented toward operationally sound solutions and participatory engagement and focuses on public health needs and solidarity.

In this regard, the main value of this study was to provide an overview with a focus on transparency as an instrument to increase the State’s management capacity to manage policies. However, this will not happen spontaneously. The availability of data and information alone is not enough to improve governance, it is necessary to counterbalance the hegemony of power in the industry.

## Data Availability

The original contributions presented in the study are included in the article/supplementary material. Further inquiries can be directed to the corresponding author.

## Footnotes

1
WHO (2018a) Pharmaceutical system refers to the relationship/interactions between the various actors of the pharmaceutical sector, the rules that govern these interactions, and the way decisions are made, in particular in the government.
